# Effects of a Nanoencapsulated *Moringa* Leaf Ethanolic Extract on the Physiology, Metabolism and Reproductive Performance of Rabbit Does during Summer

**DOI:** 10.3390/antiox10081326

**Published:** 2021-08-23

**Authors:** Nagwa I. El-Desoky, Nesrein M. Hashem, Antonio Gonzalez-Bulnes, Ahmed G. Elkomy, Zahraa R. Abo-Elezz

**Affiliations:** 1 Department of Animal and Fish Production, Faculty of Agriculture (El-Shatby), Alexandria University, Alexandria 21545, Egypt; enagwa278@gmail.com (N.I.E.-D.); ahmed.elkoumi@alexu.edu.eg (A.G.E.); n.ebrahim10@yahoo.com (Z.R.A.-E.); 2 Departamento de Produccion y Sanidad Animal, Facultad de Veterinaria, Universidad Cardenal Herrera-CEU, C/Tirant lo Blanc, 7, 46115 Valencia, Spain

**Keywords:** *Moringa* leaf extract, nanoencapsulation, rabbit, physiology, reproduction

## Abstract

This study investigated the effect of *Moringa* leaf ethanolic extract (MLEE) on heat-tolerance variables and the reproductive performance of rabbit does bred under hot climate conditions. Additionally, the effect of nanoencapsulation technology on the biological efficiency of MLEE was considered. A total of 56 rabbit does were randomly divided into four experimental groups and treated with 50 mg/kg body weight (BW) nonencapsulated MLEE, 25 or 10 mg/kg BW nanoencapsulated MLEE, or not treated (Control, C). The treatments continued for 50 days, including mating and pregnancy times. Physiological and hematochemical variables, hormonal profiles, and reproductive performance (kindling rate and litter characteristics) were determined. The active components of MLEE were identified. The results indicated that MLEE has 30 active components. All MLEE-based treatments reduced heat-stress-related indicators, such as rectal temperatures, respiratory rates and heart rate; improved hematochemical attributes, redox status, and hormones (progesterone and prolactin); and increased the total litter size, the kindling rate, litter size at birth and litter weight at birth. Adding MLEE can alleviate the negative impacts of heat stress by improving metabolism, redox status, and hormonal balance during pregnancy. These effects were seen whether MLLE was in free or encapsulated forms. However, the use of nanoencapsulated MLEE allowed 80% reduction (10 mg/kg BW) in the optimal dose (50 mg/kg BW) without affecting the efficiency of the treatment. These results support the importance of nanoencapsulation technology in improving the bioavailability of active components when they are orally administered.

## 1. Introduction

Rabbits are a good alternative to larger mammals for meat production and, thus, an adequate alternative source for the increasing population in developing countries [1]. Rabbits are characterized by high reproductive performance compared to that of other farm animals; unfortunately, they are sensitive to heat stress, which compromises productive rate and economic spin-off [2]. Increasing global warming represents a real challenge to the rabbit industry, particularly in arid and semi-arid regions; the breeding season could be restricted for several months due to the negative impacts of heat stress (high ambient temperatures) on fertility. Hence, it is necessary to investigate strategies for mitigating the consequences of heat stress on the production of rabbits.

Rabbit does are more susceptible to injury due to these effects as they exhibit critical physiological and hormonal changes during their reproductive cycles [3,4].The search for safe and effective naturally occurring feed supplements is considered to be a promising solution that can be applied to improve the harmful effects of heat stress. Phytochemicals are natural bioactive and non-nutritive plant chemicals that positively affect health [5,6,7].

One of the most effective sources of phytochemicals is the *Moringa* tree; it has several phytogenic bioactive compounds with antioxidant, antimicrobial and immunomodulatory activities that can improve the productive and reproductive performance and health of animals. Thus, this plant has gained considerable attention to be used as a feed additive or feedstuff in the livestock industry [8,9,10,11,12,13]. At the practical level, the use of a phytogenic substance that originated from active components is restricted by the stability of these molecules in the gastrointestinal tract (GIT), absorption, cellular uptake, and stability during handling and storage [14,15,16]. The nanoencapsulation of phytogenic active components is a current research need because it can be used to solve the previously mentioned problems related to the use of phytogenic components [17]. Accordingly, this study was designed to assess the effects of a non- or nanoencapsulated *Moringa* leaf ethanolic extract (MLEE) on reproductive performance, hematological variables, hormones, blood plasma metabolites, and the antioxidant indicators of rabbit does bred under environmental heat stress.

## 2. Materials and Methods

This study was conducted at the Laboratory of Rabbit Physiology Research, Agricultural Experimental Station, Faculty of Agriculture, Alexandria University, Egypt (31° 20′ N, 30° E); animals were cared for according to International Council for Laboratory Animal Science (ICLAS; http://iclas.org/members/member-list (accessed on 12 Aug 2021)).

### 2.1. Plant Extaction and Identification of Active Components

*Moringa (Moringa oleifera*) leaves were dried naturally until they reached approximately 90% dry matter and then were milled through a 1 mm screen. Moringa leaf powder (25 g/100 mL) was extracted using a 70% hydroethanolic solution at 40 °C for 72 h. The extract was filtered with Whatman No. 1 filter paper (Camlab, Cambridge, UK). The collected filtrate was evaporated to complete dryness at 45 °C. The residues were then stored at −20 °C pending use. The collected filtrate was evaporated to complete dryness at 45 °C [11]. The residues were then stored at −20 °C pending use.

Chemical constituents of MLEE were determined using gas chromatography/mass spectrometry (GC-MS) (Thermo Scientific TRACE-1300 series GC; Thermo Fisher Scientific Inc., Austin, TX, USA), fitted with a fused silica DB-5 capillary column (30 m × 0.32 mm id, 0.25 μm film thickness; Thermo Fisher Scientific Inc.) and coupled to a Triple Quadrupole Mass (TSQ 8000 Evo; Thermo Fisher Scientific Inc.). The column oven temperature was initially held at 50 °C and then increased by 5 °C/min to 250 °C, held for 2 min and then increased to the final temperature, 300 °C, by 30 °C/min and held for 2 min. Splitless injection mode (0.5 μL of a 1:1000 methanol solution) was used. The carrier gas was helium at a constant flow rate of 1 mL/min. The injector and detector temperatures were 250 °C and 290 °C, respectively. Mass spectra were scanned in the range 40 to 700 amu; the scan time was 5 scans/s. The constituents were identified by the combination of retention index data and mass spectra data using the NIST 14 mass spectral database.

### 2.2. Fabrication and Characterization of a Moringa Extract and Sodium Alginate Nanocomplex

The dried MLEE was used for the fabrication of a sodium alginate nanocomplex using calcium chloride (CaCl_2_) as a cross-linking agent by adopting the ionic-gelation method [18]. Under continuous magnetic stirring, the MLEE (1.5 g) was first mixed with the sodium alginate solution (1%, *w/v*). Then, the mixture was added dropwise using a syringe into a CaCl_2_ solution (2.2 mol/L) with a ratio of 2 sodium alginate and MLEE mixture:1 CaCl_2_ solution. The synthesized nanoparticles were centrifuged at 8000 rpm for 20 min, and the resultant nanoparticles were collected and stored at −80 °C. The physicochemical characteristics, the size, polydispersity (PdI) and zeta potential of the sodium alginate-CaCl_2_ nanoparticles conjugated or not with the extract was measured using a Scientific Nanoparticle Analyzer (Zetasizer Nano ZS, Malvern Instruments Ltd., Worcestershire, UK) at 25 °C.

The encapsulation efficiency (EE, %) of sodium alginate-CaCl_2_ nanoparticles for the MLEE was estimated by determining the phenolic content of the raw MLEE (before encapsulation, C raw) and of the resultant supernatant following the collection of the nanocomplex particles (C supernatant), using the following equation: EE (%) = C raw−C supernatant/C raw × 100. The concentrations of total phenolic compounds in the raw MLEE and the supernatant were colorimetrically determined using the Folin–Ciocalteu assay at a 765 nm absorbance wavelength and with gallic acid (GA) as a standard [19]. The concentration of total phenolic compounds was 47.60 and 20.26 µg GA equivalent/gm for raw MLEE and the supernatant, respectively.

### 2.3. Animal Husbandry and Experimental Design

The rabbit breed used in this study was of V-line breeding, a maternal synthetic line selected based on pregnancy (Department of Animal Science, Universitat Politecnica de Valencia, Valencia, Spain; [20]). Fifty-six nulliparous (six-months-old) female rabbits, weighing 2.75 ± 0.18 kg, were housed in a naturally ventilated building under similar management and hygiene conditions. Rabbit does were kept in individual galvanized wire cages (60 L × 55 W × 40 H cm). Batteries were equipped with feeders for pelleted rations and automatic drinkers. Freshwater was available ad libitum. Rabbit does received their daily maintenance according to the National Research Council (NRC, [21]). The ingredients and chemical analysis of the experimental diet are shown in Table 1. Rabbit does were randomly divided into four experimental groups and treated with 50 mg/kg BW nonencapsulated MLEE (FM_50_), 25 mg/kg BW nanoencapsulated MLEE (NM_25_), 10 mg/kg BW nanoencapsulated MLEE (NM_25_) or not treated (C). Rabbit does orally received different treatments for 50 d, starting from 20 d before insemination to the entire pregnancy period (30 d).

### 2.4. Metrological Data

Ambient temperature (°C) and relative humidity (RH, %) were recorded daily inside the rabbitry using an electronic digital thermo-hygrometer. The overall mean of the maximum and minimum temperatures, RH (%) and the temperature–humidity index (THI) during the experimental period is estimated. The temperature–humidity index (THI) was calculated according to the following equation: THI = db °C–[(0.31–0.31 × RH%) × (db °C–14.4)], where db °C = dry bulb temperature in Celsius, and RH% = percentage of relative humidity. The THI values were classified as the absence of heat stress (<27.8), moderate heat stress (27.8–28.8), severe heat stress (28.9–29.9) and very severe heat stress (>30.0) for rabbits, according to the classification of Marai et al. [23].

### 2.5. Physiological Parameters

Each rabbit doe was weighed weekly in the morning before feed was offered. The feed intake was calculated weekly by subtracting the unconsumed feed from the total amount offered and recorded as g/day. Rectal temperature was individually measured in the morning using a digital thermometer gently introduced into the rectum and attached to the rectal wall until a fixed reading was obtained. The respiration rate was measured by counting the number of flank movements during the complete inhalation–exhalation cycle (breath) per minute [24]. The heart rate was measured by counting the number of beats per minute using a *stethoscope* [23]. These data were used to estimate the mean of each variable during premating (day −10), mating (day 0) and days 10, 17 and 24 of pregnancy.

### 2.6. Hematochemical Variables and the Hormonal Profile

Blood samples were collected from the marginal ear vein of each rabbit using heparinized tubes during premating (day −10), mating (day 0) and days 10, 17 and 24 of pregnancy [25]. Each blood sample was divided into two subsamples: the first subsample was used to assess hematological variables, and the second subsample was centrifuged at 2000× *g* for 20 min at 4 °C to obtain plasma. The plasma samples were stored at −20 °C for further analyses. The counts of red blood cells (RBCs), packed corpuscular volume (PCV) and hemoglobin (Hb) were determined in the first subsamples (whole blood). The concentration of blood Hb was assessed by the colorimetric method using commercial kits (Reactivos GPL, Barcelona, Spain). Blood plasma metabolites, including total protein, albumin, glucose, cholesterol, triglycerides, activities of alanine aminotransferase (ALT) and aspartate aminotransferase (AST), were determined using the colorimetric method with commercial kits (BioSystem SA, Barcelona, Spain). Additionally, the total antioxidant capacity (TAC) and reduced glutathione enzyme (GSH-Px) activity were also determined as indicators of the antioxidant status of plasma (Biodiagnostic, Giza, Egypt). Progesterone and prolactin concentrations were analyzed in blood plasma samples collected during pregnancy (days 10, 17 and 24) using commercial solid-phase enzyme immuno-assay ELISA kits obtained from Pointe Scientific Inc., MI, USA and Cloud-Clone Corp, TX, USA, respectively. The methods’ sensitivity was 0.0625 ng/mL and 0.063 ng/mL for progesterone and prolactin, respectively. The corresponding intra-assay and inter-assay coefficients of variation were 2.4–2.6% and <10%–<12%, respectively.

### 2.7. Productive and Reproductive Performance

After 20 days from starting of the treatment, does were mated with previously evaluated fertile bucks. The kindling rate ([number of delivered does/number of mated does] × 100), litter size at birth (total rabbits born, alive and dead) and litter weight at birth were recorded [24].

### 2.8. Statistical Analyses

All the statistical analyses were carried out using the Statistical Analysis Software package (SAS, Version 8. Cary, NC, USA: SAS Institute; 2001). The MIXED procedure for repeated measurement was used to assess the fixed effects of treatment (C, FM_50,_ NM_25_ and NM_10_), status (physiological status at time of sampling and/or data collection) and the treatment by status interaction on physiological and hematochemical variables, redox status, hormonal profiles and reproductive performance. The rabbit dose effect was introduced as a random factor in this model. One-way ANOVA was used to assess the treatment effects on litter size, litter viability and litter weight, while a Chi-square test was used to assess the effects of treatments on the kindling rate. Differences among treatment means were tested by Duncan’s multiple range tests. All the results are presented as the least square mean (±SEM), with significance accepted at *p* < 0.05.

## 3. Results

### 3.1. Chemical Compositions and Characterization of a Moringa Extract and Sodium Alginate Nanocomplex

The analysis of MLEE identified 30 chemical compounds belonging to different chemical families and with different biological activities. Among the detected chemical compounds, the abundant compounds were phytosphingosine (10.46%), N-acetylneuraminic acid,2,3-dehydro-2-deoxy- (6.50%), nialamide (5.45%), 1-Stearoyl-2-oleoyl-sn-glycero-3-phosphoethanolamine (4.83%), leukotriene E4 methyl ester (4.45%), D-erythro-sphingosine C-20 (4.31%) and exo-norbornyl alcohol (4.22%) (Table 2). Other 22 compounds were detected with a range of <4.0 to <1% (Table 2). The physicochemical characterization of alginate–CaCL_2_ nanoparticles and alginate–CaCL_2_ nanoencapsulated MLEE revealed that the mean size was 195.10 and 93.69 nm; the zeta potential was −3.41 and 8.95 mV; and PdI was 0.457 and 0.442, respectively. The encapsulation efficiency of alginate–CaCL_2_ nanoparticles for MLEE was 57.43% (Table 3).

### 3.2. Metrological Variables

The means of ambient temperature, relative humidity, THI and daylight length (photoperiod) during the experimental period were 31.67 °C ± 1.21 °C, 82.67% ± 2.92%, 30.73 ± 1.23 and 13.66 h ± 0.14 h, respectively (Table 4).

### 3.3. Physiological Variables

**Figure 1:**
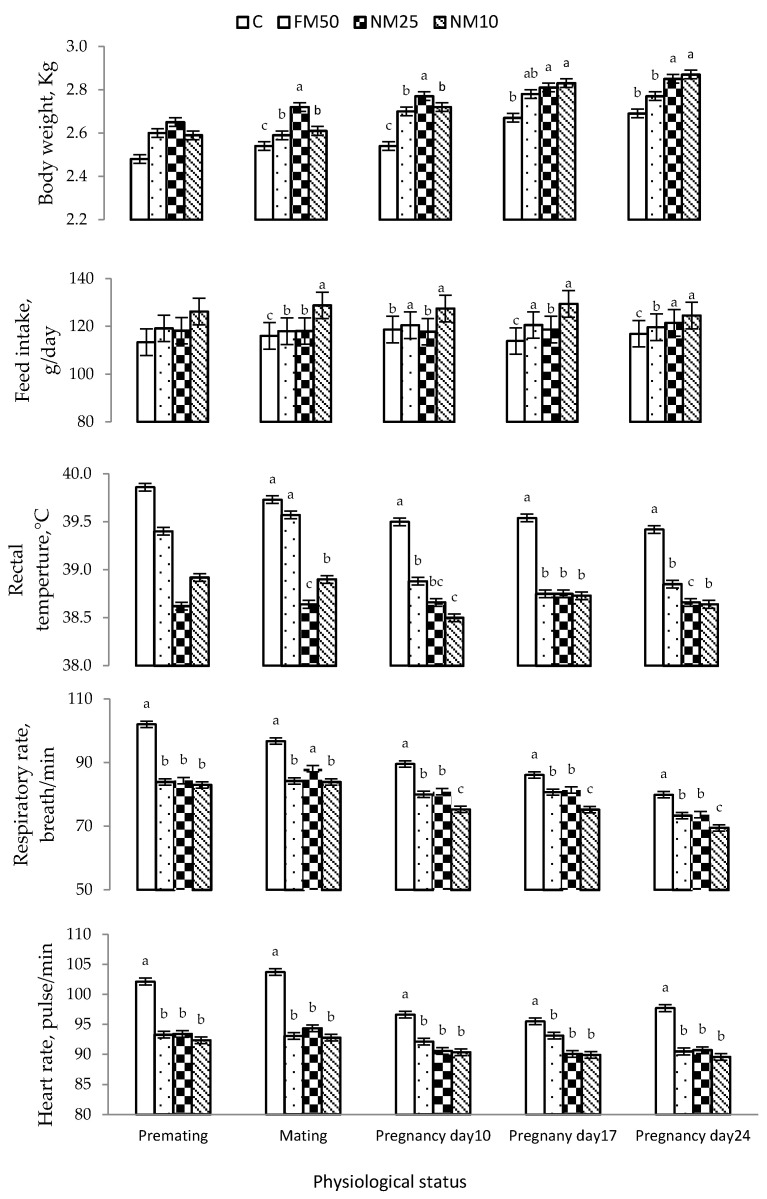
Mean (± SEM) treatment by physiological status effects on bodyweight, feed intake, rectal temperature, the respiratory rate and the heart rate of female rabbits. C = 0 mg/kg BW MLEE, FM_50_ = 50 mg/kg BW MLEE, NM_25_ = 25 mg/kg BW nanoencapsulated MLEE and NM_10_ = 10 mg/kg BW nanoencapsulated MLEE. Means within the same physiological status having different superscripts (a, b, c) differ significantly (*p* < 0.05).

The effects of different treatments (C = 0 mg/kg BW MLEE, FM_50_ = 50 mg/kg BW MLEE, NM_10_ = 10 mg/kg BW nanoencapsulated MLEE and NM_25_ = 25 mg/kg BW nanoencapsulated MLEE) on BW, feed intake, rectal temperature, respiratory rate and heart rate are presented in Table 5 and Figure 1. Compared to C treatment, all other treatments significantly increased the BW and feed intake of does. Through the experimental period, FM_50_, NM_10_ and NM_25_ significantly decreased rectal temperature, the respiratory rate and the heart rate compared to C treatment, and the lowest values were observed in NM_10_ (Table 5 and Figure 1).

### 3.4. Blood Plasma Metabolites

**Figure 2:**
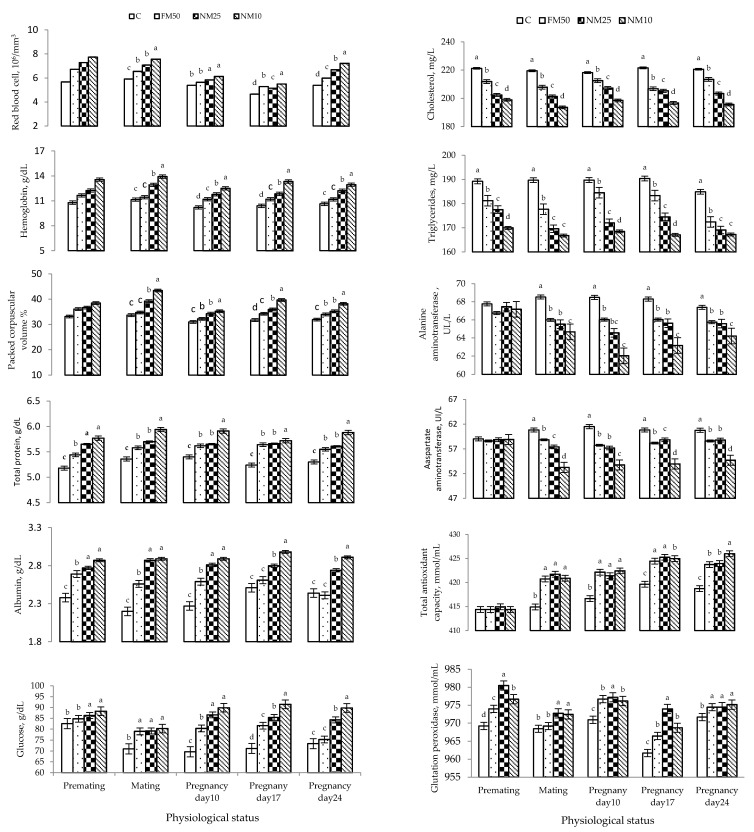
Changes (means ± SEM) in the blood plasma metabolites of rabbit does during the experimental period. C = 0 mg/kg BW MLEE, FM_50_ = 50 mg/kg BW MLEE, NM_25_ = 25 mg/kg BW nanoencapsulated MLEE and NM_10_ = 10 mg/kg BW nanoencapsulated MLEE. Means within the same physiological status having different superscripts (a, b, c, d) differ significantly (*p* < 0.05).

**Figure 3:**
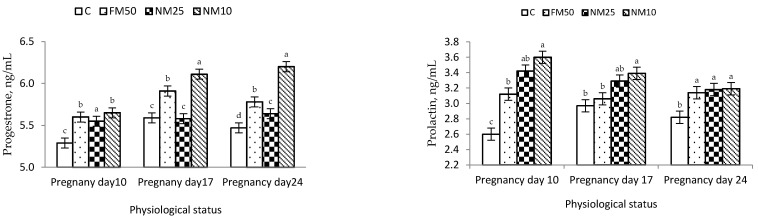
Changes (means ± SEM) in the blood plasma progesterone and prolactin of rabbit does during pregnancy. C = 0 mg/kg BW MLEE, FM_50_ = 50 mg/kg BW MLEE, NM_25_ = 25 mg/kg BW nanoencapsulated MLEE and NM_10_ = 10 mg/kg BW nanoencapsulated MLEE. Means within the same physiological status having different superscripts (a, b, c, d) differ significantly (*p* < 0.05).

The effects of different treatments (C = 0 mg/kg BW MLEE, FM_50_ = 50 mg/kg BW MLEE, NM_10_ = 10 mg/kg BW nanoencapsulated MLEE and NM_25_ = 25 mg/kg BW nanoencapsulated MLEE) on the blood plasma of female rabbits during the experimental period are presented in Table 6 and Figure 2. Compared with the C treatment, all MLEE-based treatments significantly increased hematological variables, including RBC, Hb and PCV. The highest values were observed for a low concentration of encapsulated MLEE (NM_10_). The same trend was observed for blood plasma metabolites (total protein, albumin and glucose), whereas all MLEE-based treatments significantly decreased the blood plasma concentrations of cholesterol, triglycerides, ALT and AST. All MLEE-based treatments significantly increased the TAC and GSH-Px compared to the C treatment, and the highest value was observed for the NM_10_ treatment. Both levels of nanoencapsulated MLEE treatments significantly increased blood plasma progesterone and prolactin compared to those due to the C treatment, whereas nonencapsulated MLEE resulted in intermediate values (Table 6 and Figure 3).

### 3.5. Reproductive Performance

The effects of different treatments (C = 0 mg/kg BW MLEE, FM_50_ = 50 mg/kg BW MLEE, NM_10_ = 10 mg/kg BW nanoencapsulated MLEE and NM_25_ = 25 mg/kg BW nanoencapsulated MLEE) on the reproductive performance of rabbit does are presented in Table 7. Rabbit does that received the NM_10_ treatment had the highest kidding rate, followed by those that received the FM_50_ and NM_25_, compared with those that received the C treatment. All other treatments significantly increased total litter sizes and viable litters at birth (Table 7). Compared with all treatments, the NM_10_ treatment significantly increased the litter weight at birth.

## 4. Discussion

This study is the first devoted to innovating a phytogenic feed additive using nanotechnology approaches in the livestock field. The development of encapsulation techniques facilitates the protection, as well as the controlled and targeted release, of bioactive molecules applied in the pharmaceutical, nutraceutical and food industries to improve their bioavailability (absorption and cellular intake) and to enhance the stability of bioactive compounds during processing and storage processes [14,15]. For this purpose, many natural polymers are used to encapsulate different bioactive components, including phytogenic crude extracts, used as natural-functioning nutritional supplements with health benefits [17,26]. This study used the nanoencapsulation ionic-gelation method for innovating a new feed additive that could be used in the rabbit industry during periods of heat stress, aiming at the phytochemicals of *Moringa* leaf extracts, specifically polyphenols. Based on several previous studies, a *Moringa* leaf extract has several phytogenic bioactive compounds with antioxidant, antimicrobial and immunomodulatory activities that can improve animals’ reproductive performance and health [10,11,13].

In this study we used sodium alginate as a natural polymer for the nanoencapsulation process to ensure the safety of the final product to animal and human health. This natural polymer has been used as an efficient coating material with efficient protection ability due to its high encapsulation efficiency for active components of plant extracts, mainly phenolic compounds [27]. Alginate is a natural anionic polyelectrolyte polymer that encompasses unbranched binary copolymers of (1–4) linked D-mannuronic acid (M) and L-guluronic acid (G) residues with widely varying composition. Alginate is extracted from different species of brown algae (*Phaeophyceae*). Based on the unique physicochemical properties (biodegradability, biocompatibility and capability of forming three-dimensional gels in the presence of divalent cations such as CaCl2), low cost and simplicity of use, this polymer is considered one of the suitable choice materials for the encapsulation process [18].

The encapsulation technique used in this study was efficient to entrap approximately 57% of the bioactive compounds of the *Moringa* leaf extract, as indicated by the encapsulation efficiency of the *Moringa* extract phenolic compounds. The ionic-gelation method is an efficient and low-cost encapsulation technique that does not require specialized equipment, high temperature or organic solvents, making it suitable for encapsulating hydrophobic or hydrophilic compounds[15].

In this study, the conjugation of MLEE with alginate–CaCl_2_ nanoparticles allowed an 80% reduction in dose with satisfactory positive effects on the reproductive performance of female rabbits. These results agree with those obtained in previous studies, aiming to encapsulate active phytogenic compounds, mainly polyphenols. This method was rapid and easily adapted to the industrial scale [28]. For example, the aqueous leaf extract of *Stevia rebaudiana Bertoni*, which is rich in phenolic compounds, was successfully encapsulated in CaCl_2_ beads and showed high encapsulation efficiency (>60%) [18,28] values, as well as satisfactory antioxidant storage stability. Furthermore, Calvo et al. [29] found that using alginate–CaCl_2_ as a nanocarrier for antioxidant compounds (betacyanin and polyphenols) derived from beetroot industrial wastes was efficient in encapsulating (between 20% and 40%) these active components, with good conservation of the antioxidant activity (up to 70%).

These findings support the relevance of the technique used to fabricate MLEE-conjugated alginate–CaCl_2_ nanoparticles with physicochemical characteristics (<100 nm), allowing better bioavailability for target sites.

For oral delivery into the GIT (*oral pathway*), particle uptake in the GIT depends on diffusion and accessibility through mucus and contact with the cells of the GIT. The smaller particle diameter is fast diffused through GIT mucus to reach intestinal lining cells, followed by uptake through the GIT barrier to reach the blood [30]. Existing evidence indicates that particles smaller than 100 nm are absorbed in various tissues and organs. Smaller particles capable of being taken up by the villus epithelium may directly enter the bloodstream and then be predominantly scavenged by the liver and the spleen [31].

Heat stress induces various biological reactions and behavioral changes to cope with high ambient temperature and maintain thermal homeostasis [3,14]. Under heat stress, female rabbits express a high respiratory rate and water intake and low feed intake as adaptive mechanisms for high ambient temperature, which may negatively affect rabbit does’ reproductive performance if maintained for a long time [32,33]. For example, exposure of New Zealand rabbits to 41 °C led to an 18% decrease in RBC count; 20%, hemoglobin content; 22%, blood platelet count; 11.2%, total protein; 24%, albumin, and 21%, globulin [4]. Under intensive production systems, as in most rabbit farms, these biological and behavioral responses could be more challenging when animals are housed in cages rather than in natural environments [34]. Furthermore, heat stress may be a threat to females, specifically during sensitive reproductive windows, such as mating and pregnancy periods. Female rabbits are sensitive to heat stress, which is considered an important factor influencing their reproduction, fertility and physiological traits [4,35]. Overall, heat stress and accompanying elevated oxidative stress increase the risk of spontaneous abortion and reduce milk production; litter size and litter performance; and the longevity, welfare, and health status of females.

This study was conducted during the summer, when THI was 29.20, which is classified as severe heat stress for rabbits. Notably, MLEE significantly reduced the rectal temperature of female rabbits in the treated groups. Treatment with MLEE in either free or encapsulated form around mating time and pregnancy reduced heat-stress-related indicators, such as rectal temperatures, respiratory rates and heart rates. It also improved hematochemical attributes (RBC, Hb, PCV, total protein, albumin and glucose), redox status (TAC and GSH-Px) and hormones (progesterone and prolactin) and decreased cholesterol, triglycerides, ALT and AST, suggesting an improvement in the heat tolerance of the animals. These findings support the protective role of MLEE against the negative impacts of heat stress. Several mechanisms could mediate these effects, which could be due to several biologically active phytogenics in MLEE [11,13,36]. Notably, the enhancements in physiological events in MLEE-treated groups were not associated with low feed intake as one of the adaptive behavioral mechanisms for heat stress. This effect is essential for animals during their reproductive cycles, especially mating and lactation, to maintain adequate performance. Under heat stress, animals decrease feed intake to reduce metabolic heat production, leading to changes in energy balance and nutrient availability and affecting reproductive cyclicity, pregnancy and fetal development [37,38]. Given that active components of MLEE, N-acetylneuraminic acid 2,3-dehydro-2-deoxy- (6.50%) were identified in our extract, this component is a sialylated glycoprotein that has prebiotic properties, promotes neurodevelopment and boosts immune function and gut maturation [39,40]. Additionally, our MLEE has many active components with antimicrobial/anticoccidial activity, including salinomycin [41], 2- furoic acid [42], 9S,11,15 S-trihydroxythrombox-13E-enoic acid [43], rifabutin [44] and lactacystin [45]. Furthermore, active components can be used directly as valuable nutrients or to improve digestion (glycine, aminomethyl propanol, glycan le-a trisaccharide, N-acetyl leucyl-leucyl-methioninal and deoxycholic acid) or boost the energy status of animals (adenosine 5′-diphosphate and thiocyanic acid, L-arabinitol and D-gluconic acid) [46,47,48,49]. These components in MLEE explain the positive effects of the extract on digestion, the gut-intestinal microbiota ecosystem, nutrient availability and thus, the maintenance of the body weight of MLEE-treated does during pregnancy. These findings agree with that previously reported for *Moringa* plants having several nutrients that stimulate growth and increase the bioavailability of the nutrients and feed use, such as high-quality protein, vitamins, minerals, antioxidants and cytokine-type hormone antioxidant components, particularly vitamin C [12,13,50].

In this study, compared with the control, all MLEE-based treatments significantly increased hematological variables, including RBC and hemoglobin. The administration of MLEE improved redox status (higher TAC and GSH-Px), metabolism (higher energy-yielding nutrients: glucose; protein: total protein and albumin) and liver functions (ALT and AST) of does during mating and pregnancy.

Hemoglobin plays a vital role in carrying approximately 98% of oxygen throughout the animal body system, whereas PCV measures the proportion of blood made up of cells [51]. These findings might explain the improved heat-stress tolerance and maintain the rectal temperature of MLLE-treated does, without changes in respiration rates and heart rates by improving the oxygen delivery to different organs [4]. Furthermore, improved metabolism and the redox status of MLEE-treated does play vital roles in maintaining homeothermy during heat stress through different mechanisms. For example, albumin has osmoprotective properties in plasma, which regulates water balance and maintains protein and enzyme stability [52]. Moreover, glucose availability provides an easy energy source to all body cells without the need for sophisticated catabolic and/or anabolic processes that can be combined with the rise of heat, thus increasing heat stress [11]. The enhancements of the hematobiochemical attributes and redox status of MLEE-treated does could be attributed to the presence of some specific active components that stimulate the synthesis of some proteins/enzymes. For example, the increase in erythrocytes and hemoglobin concentration may be related to the effect of amino acids, vitamins [36,52] and minerals, particularly iron. Furthermore, a compound, such as ethidimuron, identified in our MLEE, acts as a precursor of intracellular glutathione. This is in line with some components’ free radical scavenging activity, such as diapocynin [53]. These results explain the improved redox status of MLEE-treated does in our study.

Rabbit does are sensitive to heat stress, which is considered to be an important factor influencing their fertility and has negative effects on their reproductive and physiological traits [3,32,54,55]. In hot climates, the breeding of rabbits is stopped in most rabbit farms due to low reproductive performance and associated health problems. In this study, MLEE-treated does showed pronounced enhancements in sex hormones and reproductive performance compared with the control. These enhancements could be ascribed to the improved metabolism and health status of does around mating time and during pregnancy, as discussed earlier. In this context, some active components in MLEE positively affect specific reproductive events and functions. Sphingolipids, such as phytosphingosine (assembling 10.46% in our MLEE), are signaling molecules that regulate various physiological activities. In a study on pigs, exogenous phytosphingosine-1-phosphate (P1P) administration influenced animal reproduction by increasing porcine oocyte maturation and preimplantation embryo development through the regulation of oxidative stress and apoptosis signaling pathways [56]. P1P treatment upregulated the gene expression involved in cumulus expansion (Has2 and epidermal growth factor), antioxidant enzymes (superoxide dismutase and catalase) and developmental competence (octamer-binding transcription factor 4 (Oct4)) while activating extracellular signal-regulated kinase1/2 and (serine/threonine) protein kinase B signaling. P1P treatment also influenced oocyte survival by shifting the ratio of B-cell lymphoma 2 to Bax while inactivating (C-Jun N-terminal kinase) signaling. Other components, such as phosphatidylethanolamine (phospholipid depravities) and palmitic acid, play a vital role in cell membrane integrity. They act as structural components in a bilayer cell membrane [57,58]. Furthermore, nailamaid with an antidepressant activity[59]; folinic acid with antianemia, anticancer, anti-inflammatory, antitoxic activities[60]; and leukotriene E4 methyl ester [61] with anti-inflammatory and immunomodulatory effects were identified in MLEE. These components might attenuate the harmful effects of some inflammatory factors’ production, such as histamine and prostaglandins, which might negatively affect reproductive performance [62,63,64]. These could be confirmed in our study, as kindling rates and litter sizes were significantly higher in MLEE-treated does. The enhancements in these two variables reflect the enhancement in oocyte quality, the fertilization rate and embryo survival through improved cell membrane integrity.

Finally, MLEE exerted positive effects on progesterone and prolactin levels during pregnancy. These results partially follow those obtained by Ajuogu et al. [65]. The addition of a 15 mg/kg leaf powder of *Moringa* significantly improved progesterone concentrations but did not affect prolactin concentrations during pregnancy. The authors suggested that *Moringa* leaves may contain active components that can directly act on the uterus and ovary, interfering with the release of prostaglandins [65]. The high level of progesterone during the first half of pregnancy plays a crucial role in preparing the uterus for embryo implantation by suppressing uterine motility/contractions during the first few days of pregnancy, thus increasing the opportunity for pregnancy maintenance [24]. In rabbits, the levels of prolactin hormone increase from 3–4 d of gestation and remain elevated for 15–21 d of gestation. Such an increase is essential to maintain adequate concentrations of progesterone during pregnancy [66].

Notably, the weights of litters born for encapsulated MLEE-treated does were higher than those recorded for control and nonencapsulated MLEE-treated does. This may refer to the higher bioavailability of MLEE components to fetuses through improved transfer via the placenta, as nanoencapsulation may facilitate the transfer of active components, specifically those with high molecular weight and low solubility, through the fetal–placental circulating system[67].

It is worth note that the low dose of nanoencapsulated MLEE (NM_10_) resulted in better responses, as indicated by most determined variables, than the high dose of nanoencapsulated MLEE (NM_25_). This could be due to the improved bioavailability of some active components, which, though it is an advantage of the nanoencapsulation process may interfere with the competence of some biological processes. For example, MLEE is rich with phthalate, which has endocrine-disrupting effects [11]. Moreover, increased antioxidant nutrients availability, mainly phenolic compounds and vitamins, can act as prooxidants by increasing oxidative stress [6,68].

## 5. Conclusions

This study confirms the positive role of MLEE as a supplement in heat-stress tolerance, metabolism and the reproductive performance of rabbit does bred under natural heat-stress conditions. The positive effects were due to the enrichment of MLEE with an impressive range of active components that can mend negative impacts of heat stress by improving digestion and feed use, energy and redox status, and hormonal balance during pregnancy. These effects have been seen whether MLLE was in free or in encapsulated form. However, the use of the nanoencapsulated form allowed an 80% reduction (10 mg/kg BW) in the optimal dose (50 mg/kg BW) without affecting the efficiency of the treatment. These results support the importance of nanoencapsulation technology in improving the bioavailability of active components when they are orally administered.

## Figures and Tables

**Table 1:** Basal diet ingredients and chemical analysis (expressed as g/kg dry matter, DM).

Items	Basal Diet
Ingredients	
Alfalfa hay	280
Barley	180
Wheat bran	250
Yellow corn	60
Soybean meal	180
Barley grain	10
Molasses	30
Di-calcium phosphate	10
NaCl and premix ^†^	10
Analyzed composition (on DM basis) ^‡^	
Crude protein	17.54
Ether extract	2.05
Crude fiber	12.53
Ash	9.43
Nitrogen-free extract	59.45

^†^ For each 1 kg of premix (minerals and vitamins mixture) contains: vit. A, 20,000 IU; vit. D3, 15,000 IU; vit. B1, 0.33; vit. B2, 1.0 g; vit. B6, 0.33 g; vit. B5, 8.33 g; vit. B12, 1.7 mg; pantothenic acid, 3.33 g; biotin, 33 mg; folic acid, 0.83 g; choline chloride, 200 g; vit. E, 8.33 g; and vit. K, 0.33 g. ‡ Chemical analysis according to AOAC [22].

**Table 2:** Chemical composition of a *Moringa* leaf ethanolic extract.

Retention Time, min	Compounds	Area of Component, %	Chemical Formula	Molecular Weight
20.28	Phytosphingosine	10.46	C_18_H_39_NO_3_	317
8.91	N-acetylneuraminic acid,2,3-dehydro-2-deoxy-	6.50	C_11_H_17_NO_8_	291
7.43	Nialamide	5.45	C_16_H_18_N4O_2_	298
25.75	1-Stearoyl-2-oleoyl-sn-glycero-3-phosphoethanolamine	4.83	C_41_H_80_NO_8_P	745
9.16	Leukotriene E4 methyl ester	4.45	C_24_H_39_NO_5_S	453
15.12	D-erythro-sphingosine C-20	4.31	C_20_H_41_NO_2_	327
8.59	Exo-norbornyl alcohol	4.22	C_7_H_12_O	112
24.71	Ethidimuron	3.96	C_7_H_12_N_4_O_3_S_2_	264
20.63	N-methylformamide	3.93	C_2_H_5_NO	59
3.37	Salinomycin	3.75	C_42_H_70_O_11_	750
7.77	Folinic acid	3.69	C_20_H_23_N_7_O_7_	473
11.52	Lactacystin	3.66	C_15_H_24_N_2_O_7_S	376
25.59	Methyl alcohol	3.46	CH_4_O	32
8.34	Pentadecanoic acid, 14-methyl-	3.40	C_16_H_32_O_2_	256
17.15	L-arabinitol	3.37	C_5_H_12_O_5_	152
17.83	1-Hexadecanoyl-2-(5Z,8Z,11Z,14Zeicosatetraenoyl)-sn-glycero-3-phosphoethanolamine	3.31	C_41_H_74_NO_8_P	739
18.80	Deoxycholic acid	3.11	C_24_H_40_O_4_	392
27.48	Glycan le-a trisaccharide	2.64	C_20_H_35_NO_15_	529
9.56	2-furoic acid	2.52	C_5_H_4_O_3_	112
18.07	Glycine	2.43	C_2_H_5_NO_2_	75
7.53	Diapocynine	2.20	C_18_H_18_O_6_	330
18.22	3-pentanol, 3-ethyl-	2.08	C_7_H_16_O	116
30.39	Rifabutin	1.93	C_46_H_62_N_4_O_11_	846
14.33	Palmatic acid	1.88	C_4_H_9_NO_2_	103
20.48	Amino methyl propanol	1.73	C_4_H_11_NO	89
3.25	Adenosin’ 5′-diphosphate	1.58	C_10_H_15_N_5_O_10_P_2_	427
3.2	N-acetylleucyl-leucyl-methioninal	1.46	C_19_H_35_N_3_O_4_S	401
7.08	Thiocyanic acid	1.44	CHNS	59
9.23	9S,11,15S-trihydroxythrombox-13E-enoic acid	1.32	C_20_H_36_O_6_	372
24.50	D-gluconic acid	0.94	C_6_H_12_O_7_	196

**Table 3:** Physicochemical characteristics of the alginate–calcium chloride nanoparticles and prepared nanoencapsulated Moringa leaf ethanolic extract (MLEE).

Item	Alginate–Calcium Nanoparticles	Nanoencapsulated MLEE
Size, nm	195.10	93.69
Zeta potential, mV	−3.41	8.95
Poly disparity index	0.457	0.442
Entrapment efficiency, %	-	57.43
Total phenols, eq-mg gallic acid/kg DM	-	27.34

**Table 4:** Changes in (mean ± SEM) ambient temperature (°C), relative humidity (%), temperature–humidity index (THI) and photoperiod (h) during the 10 d intervals of the experimental period (July–mid-August).

s	10	20	30	40	50	Overall	SEM	*p*-Value
Air temperature, °C	30.80 ^cd^	30.20 ^d^	31.64 ^bc^	31.80 ^b^	33.90 ^a^	31.67	1.21	<0.001
Relative humidity, %	83.50 ^a^	82.40 ^ab^	83.36 ^a^	80.90 ^b^	83.60	82.67	2.92	0.163
THI	29.95 ^bc^	29.33 ^c^	30.69 ^b^	30.77 ^b^	32.90 ^a^	30.73	1.23	<0.001
Day light length, h	14.6 ^a^	13.75 ^b^	13.47 ^d^	13.59 ^c^	13.43 ^d^	13.66	0.14	<0.001

^a,b,c,d^ Means within a row with different superscript letters are significantly different (*p* < 0.05).

**Table 5:** Effect of the nanoencapsulated *Moringa* leaf ethanolic extract (MLEE) on the physiological parameters of a female rabbit during the experimental period (mean ± SEM).

Variable		Treatment ^1^ (T) (*n* = 14/group)			SEM		*p*-Value	
	C	FM_50_	NM_25_	NM_10_		T	Status (S)	T × S
Body weight, kg	2.60 ^b^	2.69 ^a^	2.80 ^a^	2.74 ^a^	0.04	0.009	<0.001	<0.001
Feed intake, g/day	116.52 ^c^	120.35 ^b^	120.27 ^b^	126.45 ^a^	7.47	<0.001	0.02	<0.001
Rectal temperature, °C	39.67 ^a^	39.12 ^b^	38.71 ^c^	38.70 ^c^	0.04	<0.001	<0.001	<0.001
Respiratory rate, breaths/min	90.02 ^a^	78.84 ^b^	78.65 ^b^	75.71 ^c^	1.34	<0.001	<0.001	<0.001
Heart rate, pulse/min	99.15 ^a^	91.73 ^b^	91.02 ^b^	90.27 ^b^	0.64	<0.001	<0.001	<0.001

^1^ C = 0 mg/kg BW MLEE, FM_50_ = 50 mg/kg BW MLEE, NM_25_ = 25 mg/kg BW nanoencapsulated MLEE and NM_10_ = 10 mg/kg BW nanoencapsulated MLEE. Means within the same physiological status having different superscripts (a, b, c) differ significantly (*p* < 0.05).

**Table 6:** Effect of nanoencapsulated *Moringa* leaf ethanolic extract (MLEE) on the blood plasma of female rabbits during the experimental period (mean ± SEM).

Variable	Treatment ^1^ (T) (*n*=14/group)					*p*-Value		
	C	FM_50_	NM_25_	NM_10_		T	Status (S)	T × S
Hematology parameters								
Red blood cell count, 10^6^/cm^3^	5.46 ^d^	5.98 ^c^	6.40 ^b^	6.82 ^a^	0.01	<0.001	<0.001	<0.001
Hemoglobin, g/dL	10.64 ^d^	11.33 ^c^	12.20 ^b^	13.25 ^a^	0.14	<0.001	<0.001	0.04
Packed corpuscular volume, %	32.27 ^d^	34.2 ^c^	36.22 ^b^	38.96 ^a^	0.35	<0.001	<0.001	<0.001
Plasma metabolites								
Total protein, g/dL	5.30 ^d^	5.57 ^c^	5.65 ^b^	5.84 ^a^	0.04	<0.001	0.05	0.71
Albumin, g/dL	3.31 ^d^	3.52 ^c^	3.75 ^b^	3.86 ^a^	0.04	<0.001	0.49	0.51
Glucose, mg/dL	73.54 ^d^	80.20 ^c^	83.40 ^b^	87.93 ^a^	0.87	<0.001	<0.001	0.0004
Cholesterol, mg/dL	220.24 ^a^	210.50 ^b^	205.01 ^c^	196.70 ^d^	1.19	<0.001	0.32	0.10
Triglycerides, mg/dL	188.79 ^a^	179.82 ^b^	174.26 ^c^	167.91 ^d^	1.48	<0.001	<0.001	0.43
Alanine aminotransferase, IU/L	68.09 ^a^	66.11 ^b^	65.35 ^c^	63.25 ^d^	0.27	<0.001	0.32	0.36
Aspartate aminotransferase, IU/L	60.58 ^a^	58.38 ^b^	57.81 ^c^	53.51 ^d^	0.60	<0.001	0.01	<0.001
Antioxidant								
Total antioxidant capacity, mmol/mL	416.86 ^b^	421.89 ^a^	421.86 ^a^	422.13 ^a^	0.47	<0.001	<0.001	<0.001
Glutathione peroxidase, mmol/mL	968.40 ^c^	972.14 ^b^	973.82 ^ab^	975.76 ^a^	0.65	<0.001	<0.001	<0.001
Hormone								
Progesterone, ng/mL	5.45 ^c^	5.76 ^b^	6.02 ^a^	5.99 ^a^	0.03	<0.001	<0.001	<0.001
Prolactin, ng/mL	2.80 ^c^	3.11 ^b^	3.33 ^a^	3.39 ^a^	0.07	<0.001	<0.001	<0.001

^1^ C = 0 mg/kg BW MLEE, FM_50_ = 50 mg/kg BW MLEE, NM_25_ = 25 mg/kg BW nanoencapsulated MLEE and NM_10_ = 10 mg/kg BW nanoencapsulated MLEE. Means within the same physiological status having different superscripts (a, b, c, d) differ significantly (*p* < 0.05).

**Table 7:** Effect of the nanoencapsulated *Moringa* leaf ethanolic extract (MLEE) on the reproductive performance of female rabbits during the experimental period (Mean ± SEM).

Variable	Treatment ^1^ (*n* = 14/Group)				SEM	*p*-Value
	C	FM_50_	NM_25_	NM_10_		
Kindling rate, %	71.40 ^c^ (10/14)	92.85 ^b^ (13/14)	92.85 ^b^ (13/14)	100 ^a^ (14/14)	-	0.035
Litter size at birth	6.00 ^b^	6.07 ^b^	7.21 ^a^	7.57 ^a^	1.30	0.001
No. live litter sizes	5.64 ^b^	6.00 ^b^	7.07 ^a^	7.36 ^a^	1.13	<0.001
No. dead litter sizes	0.36	0.07	0.14	0.21	0.44	0.750
Litter weight at birth, g	258.18 ^b^	293.77 ^b^	316.43 ^b^	393.50 ^a^	74.93	<0.001

^1^ C = 0 mg/kg BW MLEE, FM_50_ = 50 mg/kg BW MLEE, NM_25_ = 25 mg/kg BW nanoencapsulated MLEE and NM_10_ = 10 mg/kg BW nanoencapsulated MLEE. Means within the same physiological status having different superscripts (a, b, c) differ significantly (*p* < 0.05).

## Data Availability

The data presented in this study are available on request from the corresponding author. The data are not publicly available because of privacy.
